# Supplementary material for: The potential bidirectional relationship between long COVID and menstruation
Source: Nat Commun. 2025 Sep 16;16:8187. doi: 10.1038/s41467-025-62965-7 (PMC12441152; doi:10.1038/s41467-025-62965-7)
Supplement: Supplementary file 1 — Supplementary Information [file 41467_2025_62965_MOESM1_ESM.pdf]

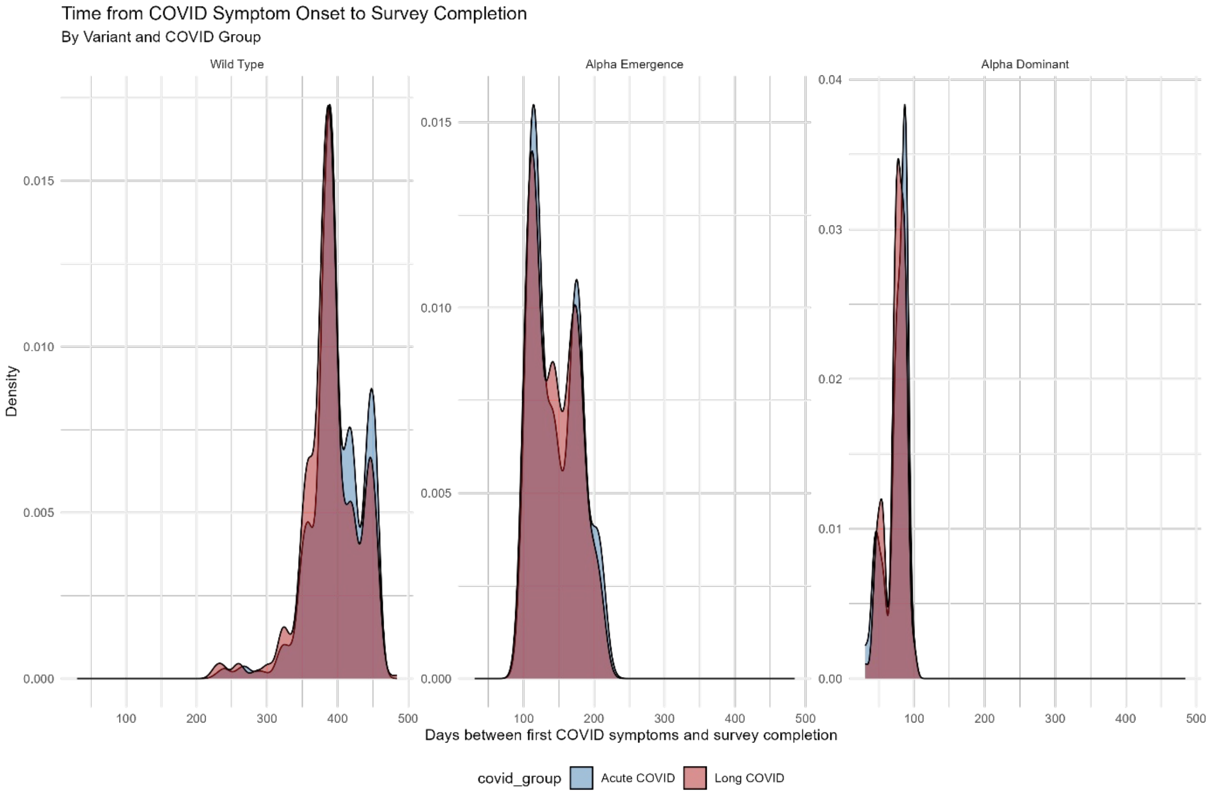

Figure S1. Distribution of time from COVID symptom onset to survey completion across variant and disease groups. To evaluate whether differences between disease groups were attributable to different COVID variants, we compared variant distributions across Long COVID and Acute COVID participants. We assigned the dominant viral variant based on symptom onset timing: Wild Type (January-August 2020, n=1,666), Alpha Emergence (September-December 2020, n=850), and Alpha Dominant (January-March 2021, n=248). Chi-square analysis revealed significant differences in variant distribution between Long COVID and Acute COVID groups ($\chi^2=37.14$, df=2, $p<0.001$), indicating differential exposure to variants between disease groups. However, when examining timing within each variant period, Wilcoxon-Mann-Whitney tests showed only minimal differences for the Wild Type variant ($p < 0.001$), with participants with acute COVID having a slightly longer time from symptom onset to survey completion (median=394 days) compared to those with Long COVID (median=389 days), representing a clinically negligible difference of 5 days. No significant timing differences were observed for Alpha Emergence ($p=0.327$) or Alpha Dominant ($p=0.140$) variants. While variant exposure differs between groups, the minimal timing differences within variants suggest this is unlikely to substantially confound disease group comparison.

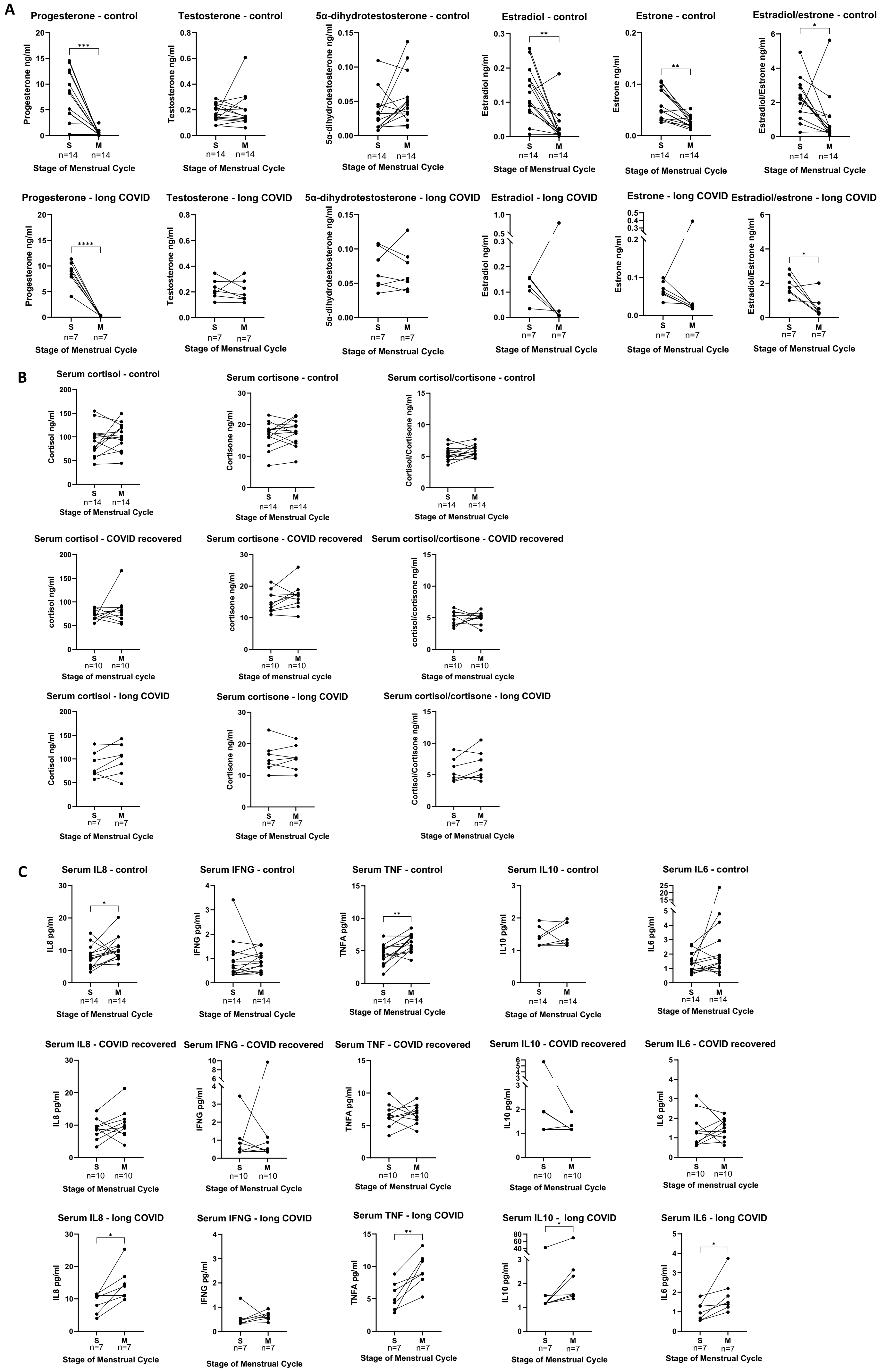

Suppl. Fig. 2. Paired secretory (S) and menstrual (M) phase serum samples from the same woman. (A) Serum ovarian hormone levels in women who have never had COVID (upper row) and those who had long COVID (lower row). In women who had never had COVID, there was a significantly lower level of serum progesterone ($p=0.0006$), estradiol ($p=0.0012$) and estradiol/estrone ratio ($p=0.0134$) in the menstrual phase compared to the secretory phase. In those with long COVID, there was a significantly lower level of serum progesterone ($p=<0.0001$) and estradiol/estrone ratio ($p=0.0469$) in the menstrual phase compared to secretory phase. (B) Serum glucocorticoids in women who have never had COVID (upper row), who had recovered from acute COVID (middle row) and those who had long COVID (lower row). (C) Serum inflammatory mediators in women who have never had COVID (upper row), those who had recovered from acute COVID (middle row) and those who had long COVID (lower row). In women who had never had COVID, there was a significantly higher level of serum IL8 ($p=0.0525$) and TNF ($p=0.0014$) in the menstrual phase compared to secretory phase. In those with long COVID, there was a significantly higher level of serum IL8 ($p=0.0166$), TNF ($p=0.0039$), IL10 ($p=0.0156$) and IL6 ($p=0.0336$) in the menstrual phase compared to secretory phase. Statistical analysis: paired t-test or Wilcoxon signed-rank test. *$p<0.05$, **$p<0.01$, ***$P<0.001$, ****$p<0.0001$.

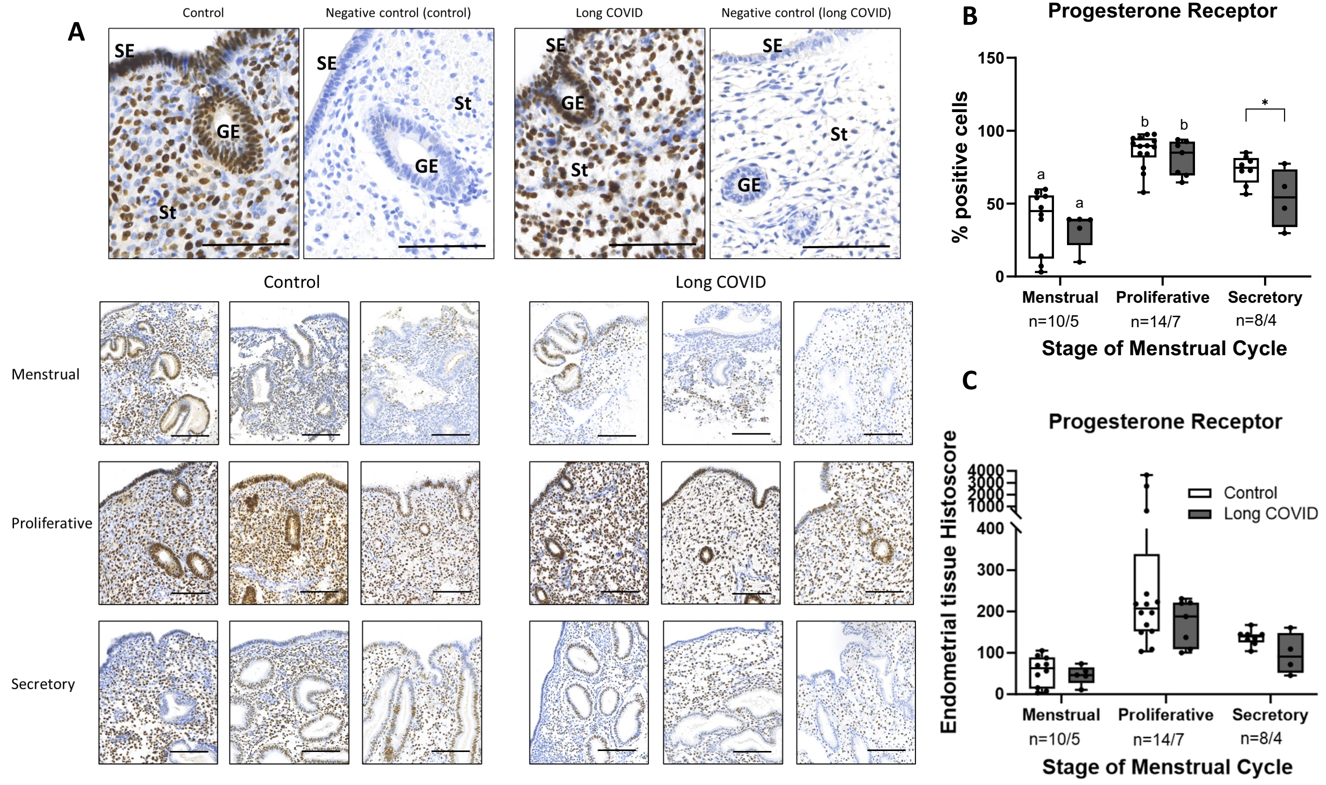

Suppl. Fig 3. Immunohistochemical staining of progesterone receptor (PGR) in endometrial tissue. (A) Representative endometrial PGR staining from women who had never had COVID (control, n=3) and those with long COVID (n=3). Upper panels are high power images and negative controls, lower panels show staining in the menstrual, proliferative and secretory phases in controls and those with long COVID. Scale bar = 100µM. (B) Quantification of the percentage of endometrial cells positive for PGR. There was a significantly lower number of cells positive for PGR in the secretory phase in those with long COVID versus controls ($p=0.0412$). (C) Histoscore (number and intensity of staining) for PGR. Box and whisker plots: box represents upper and lower quartiles with horizontal line representing the median, whiskers represent minimum and maximum values. Statistical analysis: Two way ANOVA with Tukey's multiple comparisons test: *$p<0.05$, a-b $p<0.01$.

Table S1

Pooled Multivariate Models (Cycle Frequency) _LONGCOVID

y.level	term	RRR	LowCI	HighCI	p.value	q.value
Frequent	(Intercept)	0.13	0.11	0.15	0	0
Frequent	age_scaled	1.13	1.05	1.21	0.0018	0.0088
Frequent	bmi_groupObese	1.04	0.82	1.33	0.7233	0.8777
Frequent	bmi_groupOverweight	1.01	0.82	1.24	0.9502	0.9502
Frequent	bmi_groupUnderweight	1.09	0.67	1.77	0.7314	0.8777
Frequent	covid_groupAcute COVID	1.19	1	1.43	0.0519	0.1296
Frequent	covid_groupLong COVID	1.27	1.01	1.59	0.0391	0.1247
Frequent	cyclelength_before_groupFrequent (<24 days)	13.37	11.72	15.25	0	0
Frequent	cyclelength_before_groupInfrequent (>38 days)	1.4	0.71	2.76	0.3287	0.519
Frequent	contra_past12mo_groupallCombined estrogen-progestin	1.22	1.02	1.46	0.0309	0.116
Frequent	contra_past12mo_groupallCopper IUD	1.08	0.84	1.39	0.5419	0.739
Frequent	contra_past12mo_groupallOther	1.04	0.7	1.55	0.8406	0.9339
Frequent	contra_past12mo_groupallProgestin only	1.51	1.24	1.84	1e-04	3e-04
Frequent	contra_past12mo_groupallSterilization	1.41	0.94	2.12	0.0953	0.22
Frequent	disease_before_binary_num	0.99	0.84	1.17	0.9324	0.9502
Infrequent	(Intercept)	0.02	0.01	0.03	0	0
Infrequent	age_scaled	0.95	0.79	1.15	0.6066	0.7912
Infrequent	bmi_groupObese	1.71	1.07	2.73	0.03	0.116
Infrequent	bmi_groupOverweight	0.97	0.61	1.56	0.9049	0.9502
Infrequent	bmi_groupUnderweight	2.44	1.02	5.82	0.0458	0.125
Infrequent	covid_groupAcute COVID	1.16	0.75	1.81	0.5018	0.7168
Infrequent	covid_groupLong COVID	1.5	0.87	2.56	0.1411	0.2845
Infrequent	cyclelength_before_groupFrequent (<24 days)	1.42	0.88	2.28	0.1484	0.2845
Infrequent	cyclelength_before_groupInfrequent (>38 days)	68.31	45.4	102.76	0	0
Infrequent	contra_past12mo_groupallCombined estrogen-progestin	1.35	0.9	2.04	0.1517	0.2845
Infrequent	contra_past12mo_groupallCopper IUD	0.41	0.17	0.97	0.0416	0.1247
Infrequent	contra_past12mo_groupallOther	0.36	0.08	1.59	0.1788	0.3155
Infrequent	contra_past12mo_groupallProgestin only	1.3	0.84	1.99	0.2351	0.3918
Infrequent	contra_past12mo_groupallSterilization	1.14	0.4	3.23	0.8069	0.9311
Infrequent	disease_before_binary_num	1.16	0.79	1.7	0.446	0.669

Table S2

Pooled Multivariate Models (Cycle Regularity)_LONGCOVID

y.level	term	RRR	LowCI	HighCI	p.value	q.value
Irregular (> 20 days)	(Intercept)	0.04	0.03	0.06	0	0
Irregular (> 20 days)	age_scaled	1.08	0.98	1.19	0.1502	0.267
Irregular (> 20 days)	bmi_groupObese	1.39	1.04	1.86	0.0468	0.1069
Irregular (> 20 days)	bmi_groupOverweight	0.98	0.75	1.28	0.886	0.886
Irregular (> 20 days)	bmi_groupUnderweight	1.24	0.58	2.65	0.5806	0.6407
Irregular (> 20 days)	covid_groupAcute COVID	0.98	0.75	1.27	0.8664	0.886
Irregular (> 20 days)	covid_groupLong COVID	1.19	0.84	1.71	0.3404	0.487
Irregular (> 20 days)	cycle_irreg_before_groupI did not have periods	3.68	2.4	5.62	0	2e-04
Irregular (> 20 days)	cycle_irreg_before_groupIrregular (> 20 days)	58.16	18.81	179.84	0.0018	0.0094
Irregular (> 20 days)	cycle_irreg_before_groupIrregular (10-20 days)	13.98	6.16	31.76	0.0017	0.0094
Irregular (> 20 days)	contra_past12mo_groupallCombined estrogen-progestin	0.55	0.38	0.79	0.0049	0.0173
Irregular (> 20 days)	contra_past12mo_groupallCopper IUD	0.77	0.47	1.24	0.2854	0.4567
Irregular (> 20 days)	contra_past12mo_groupallOther	0.67	0.38	1.19	0.1717	0.2891
Irregular (> 20 days)	contra_past12mo_groupallProgestin only	2.12	1.5	2.99	0.003	0.0119
Irregular (> 20 days)	contra_past12mo_groupallSterilization	1.37	0.76	2.48	0.3056	0.4656
Irregular (> 20 days)	disease_before_binary_num	1.33	1	1.76	0.0788	0.1576
Irregular (10-20 days)	(Intercept)	0.06	0.04	0.08	0	0
Irregular (10-20 days)	age_scaled	0.95	0.87	1.05	0.35	0.487
Irregular (10-20 days)	bmi_groupObese	1.5	1.17	1.93	0.0066	0.0196
Irregular (10-20 days)	bmi_groupOverweight	1.09	0.86	1.39	0.4627	0.5923
Irregular (10-20 days)	bmi_groupUnderweight	1.33	0.69	2.56	0.3966	0.5287
Irregular (10-20 days)	covid_groupAcute COVID	0.96	0.74	1.25	0.7648	0.8158
Irregular (10-20 days)	covid_groupLong COVID	1.39	1.03	1.87	0.0369	0.0909
Irregular (10-20 days)	cycle_irreg_before_groupI did not have periods	2.08	1.06	4.08	0.0746	0.1576
Irregular (10-20 days)	cycle_irreg_before_groupIrregular (> 20 days)	11.95	2.7	52.86	0.0295	0.0786
Irregular (10-20 days)	cycle_irreg_before_groupIrregular (10-20 days)	18.8	7.37	47.95	0.0026	0.0119
Irregular (10-20 days)	contra_past12mo_groupallCombined estrogen-progestin	0.63	0.47	0.86	0.0067	0.0196
Irregular (10-20 days)	contra_past12mo_groupallCopper IUD	0.87	0.57	1.32	0.5126	0.6309
Irregular (10-20 days)	contra_past12mo_groupallOther	0.6	0.33	1.09	0.0967	0.182
Irregular (10-20 days)	contra_past12mo_groupallProgestin only	2.13	1.6	2.84	5e-04	0.0038
Irregular (10-20 days)	contra_past12mo_groupallSterilization	1.21	0.63	2.35	0.5738	0.6407
Irregular (10-20 days)	disease_before_binary_num	1.08	0.84	1.39	0.5502	0.6407

Table S3

Pooled Multivariate Models (Period Flow) Long COVID

y.level	term	RRR	LowCI	HighCI	p.value	q.value
Heavier	(Intercept)	0.32	0.29	0.37	0	0
Heavier	age_scaled	0.99	0.94	1.05	0.8193	0.819273195873194
Heavier	bmi_groupObese	1.63	1.39	1.93	0	1.93901112247967e-05
Heavier	bmi_groupOverweight	1.35	1.16	1.58	0.0017	0.00174775781766423
Heavier	bmi_groupUnderweight	0.88	0.5	1.55	0.7631	0.763112496296611
Heavier	covid_groupAcute COVID	1.19	1.03	1.38	0.0538	0.0537835902556836
Heavier	covid_groupLong COVID	1.93	1.59	2.35	0	3.45990436301236e-09
Heavier	heavyperiod_beforeYes	2.22	1.99	2.49	0	0
Heavier	contra_past12mo_groupallCombined estrogen-progestin	0.87	0.75	1.02	0.1366	0.136572665227466
Heavier	contra_past12mo_groupallCopper IUD	1.25	1.02	1.54	0.0731	0.0731292438257093
Heavier	contra_past12mo_groupallOther	1.02	0.73	1.41	0.944	0.943989350702853
Heavier	contra_past12mo_groupallProgestin only	1.2	1.03	1.39	0.0493	0.0493051602000966
Heavier	contra_past12mo_groupallSterilization	1.45	1.02	2.06	0.08	0.0800367789164853
Heavier	disease_before_binary_num	1.16	1.02	1.32	0.0651	0.0651144671835299
Lighter	(Intercept)	0.27	0.24	0.31	0	0
Lighter	age_scaled	1.07	1	1.14	0.115	0.115004396531436
Lighter	bmi_groupObese	0.95	0.78	1.15	0.7631	0.763112496296611
Lighter	bmi_groupOverweight	1.1	0.92	1.3	0.4517	0.451730126299075
Lighter	bmi_groupUnderweight	0.99	0.63	1.54	0.9478	0.947818415760793
Lighter	covid_groupAcute COVID	1.1	0.93	1.31	0.4191	0.419111980115607
Lighter	covid_groupLong COVID	1.36	1.08	1.71	0.0286	0.0285771694423498
Lighter	heavyperiod_beforeYes	1.29	1.13	1.48	9e-04	0.000940631064506814
Lighter	contra_past12mo_groupallCombined estrogen-progestin	1.15	0.97	1.36	0.1819	0.18193140332603
Lighter	contra_past12mo_groupallCopper IUD	1.09	0.84	1.43	0.6597	0.659746920844253
Lighter	contra_past12mo_groupallOther	0.66	0.4	1.08	0.1612	0.161232781395383
Lighter	contra_past12mo_groupallProgestin only	1.78	1.52	2.08	0	2.66004995808089e-11
Lighter	contra_past12mo_groupallSterilization	0.92	0.57	1.51	0.813	0.813037970584817
Lighter	disease_before_binary_num	1.22	1.05	1.42	0.0308	0.0307673802773722
Lighter & Heavier	(Intercept)	0.34	0.3	0.4	0	0
Lighter & Heavier	age_scaled	0.93	0.88	0.99	0.0651	0.0651144671835299
Lighter & Heavier	bmi_groupObese	1.3	1.1	1.53	0.0084	0.00844465629066304
Lighter & Heavier	bmi_groupOverweight	1.17	1	1.38	0.1073	0.10734425179191
Lighter & Heavier	bmi_groupUnderweight	0.83	0.52	1.31	0.5929	0.592873185204011
Lighter & Heavier	covid_groupAcute COVID	1.06	0.89	1.26	0.6597	0.659746920844253
Lighter & Heavier	covid_groupLong COVID	1.57	1.26	1.96	3e-04	0.000296832760392984
Lighter & Heavier	heavyperiod_beforeYes	1.71	1.5	1.96	0	2.04124717129162e-11
Lighter & Heavier	contra_past12mo_groupallCombined estrogen-progestin	0.63	0.52	0.76	0	1.81851814923251e-05
Lighter & Heavier	contra_past12mo_groupallCopper IUD	1.04	0.83	1.31	0.8034	0.803416650724976
Lighter & Heavier	contra_past12mo_groupallOther	1.16	0.81	1.65	0.5929	0.592873185204011
Lighter & Heavier	contra_past12mo_groupallProgestin only	1.05	0.84	1.32	0.7631	0.763112496296611
Lighter & Heavier	contra_past12mo_groupallSterilization	1.09	0.71	1.65	0.7944	0.794354706883351
Lighter & Heavier	disease_before_binary_num	1.04	0.9	1.21	0.7277	0.727726266567449

Table S4

Pooled Multivariate Models (Period Duration) LONG COVID

term	PR	LowCI	HighCI	p.value	q.value
(Intercept)	0.15	0.11	0.21	0	0
age_scaled	0.74	0.64	0.86	1e-04	3e-04
bmi_groupObese	1.45	0.98	2.13	0.0679	0.1057
bmi_groupOverweight	1.07	0.62	1.84	0.816	0.816
bmi_groupUnderweight	2.12	0.77	5.8	0.1454	0.2036
covid_groupAcute COVID	1.22	0.85	1.77	0.2856	0.3332
covid_groupLong COVID	2.26	1.46	3.49	3e-04	6e-04
period_lengh_before_groupProlonged (8 days+)	136.75	27.81	672.41	2e-04	6e-04
contra_past12mo_groupallCombined estrogen-progestin	0.69	0.4	1.2	0.1963	0.2498
contra_past12mo_groupallCopper IUD	7.37	4.14	13.12	0	0
contra_past12mo_groupallOther	1.17	0.39	3.46	0.7788	0.816
contra_past12mo_groupallProgestin only	2.64	1.87	3.72	0	0
contra_past12mo_groupallSterilization	2.7	1.07	6.81	0.0352	0.0616
disease_before_binary_num	1.69	1.21	2.36	0.0022	0.0044

Table S5

Pooled Multivariate Models (Inter-Menstrual Bleeding)

y.level	term	RRR	LowCI	HighCI	p.value	q.value
Less	(Intercept)	0.02	0.01	0.03	0	0
Less	age_scaled	1.05	0.9	1.22	0.5473	0.6841
Less	bmi_groupObese	1.06	0.72	1.55	0.7622	0.8795
Less	bmi_groupOverweight	1.24	0.83	1.86	0.3054	0.4907
Less	bmi_groupUnderweight	0.62	0.16	2.39	0.4839	0.6804
Less	covid_groupAcute COVID	0.89	0.58	1.34	0.5639	0.6858
Less	covid_groupLong COVID	1.19	0.74	1.9	0.4735	0.6804
Less	cyclelength_before_groupFrequent (<24 days)	1.3	0.95	1.76	0.0972	0.2082
Less	cyclelength_before_groupInfrequent (>38 days)	3.03	1.65	5.56	3e-04	0.0013
Less	contra_past12mo_groupallCombined estrogen-progestin	1.5	1	2.25	0.0488	0.1157
Less	contra_past12mo_groupallCopper IUD	2.76	1.71	4.47	0	1e-04
Less	contra_past12mo_groupallOther	0.98	0.35	2.7	0.9659	0.9659
Less	contra_past12mo_groupallProgestin only	2.75	1.92	3.93	0	0
Less	contra_past12mo_groupallSterilization	1.49	0.64	3.5	0.3575	0.5548
Less	disease_before_binary_num	1.67	1.21	2.3	0.0016	0.0052
More	(Intercept)	0.15	0.12	0.19	0	0
More	age_scaled	0.91	0.85	0.98	0.0109	0.0326
More	bmi_groupObese	0.98	0.82	1.17	0.8057	0.9064
More	bmi_groupOverweight	1.06	0.89	1.26	0.5306	0.6841
More	bmi_groupUnderweight	0.97	0.59	1.59	0.9056	0.9261
More	covid_groupAcute COVID	1.15	0.97	1.36	0.1076	0.22
More	covid_groupLong COVID	1.59	1.29	1.97	0	1e-04
More	cyclelength_before_groupFrequent (<24 days)	1.05	0.91	1.2	0.531	0.6841
More	cyclelength_before_groupInfrequent (>38 days)	1.22	0.84	1.78	0.2916	0.4861
More	contra_past12mo_groupallCombined estrogen-progestin	1.23	1.03	1.47	0.0222	0.0554
More	contra_past12mo_groupallCopper IUD	2.67	2.08	3.43	0	0
More	contra_past12mo_groupallOther	1.29	0.89	1.88	0.1839	0.3449
More	contra_past12mo_groupallProgestin only	2.75	2.32	3.26	0	0
More	contra_past12mo_groupallSterilization	1.07	0.69	1.66	0.7551	0.8795
More	disease_before_binary_num	1.43	1.23	1.66	0	0
Sometimes less and sometimes more	(Intercept)	0.04	0.03	0.05	0	0
Sometimes less and sometimes more	age_scaled	0.94	0.83	1.05	0.2735	0.4733
Sometimes less and sometimes more	bmi_groupObese	1.12	0.82	1.52	0.4744	0.6804
Sometimes less and sometimes more	bmi_groupOverweight	1.04	0.74	1.45	0.836	0.9175
Sometimes less and sometimes more	bmi_groupUnderweight	0.95	0.43	2.11	0.9055	0.9261
Sometimes less and sometimes more	covid_groupAcute COVID	0.81	0.59	1.1	0.1784	0.3449
Sometimes less and sometimes more	covid_groupLong COVID	1.03	0.71	1.5	0.8572	0.9184
Sometimes less and sometimes more	cyclelength_before_groupFrequent (<24 days)	1.44	1.15	1.8	0.0016	0.0052
Sometimes less and sometimes more	cyclelength_before_groupInfrequent (>38 days)	1.67	0.94	2.96	0.0821	0.1847
Sometimes less and sometimes more	contra_past12mo_groupallCombined estrogen-progestin	1.43	1.06	1.93	0.0179	0.0495
Sometimes less and sometimes more	contra_past12mo_groupallCopper IUD	3.2	2.27	4.51	0	0
Sometimes less and sometimes more	contra_past12mo_groupallOther	0.55	0.2	1.51	0.2452	0.4414
Sometimes less and sometimes more	contra_past12mo_groupallProgestin only	2.88	2.21	3.77	0	0
Sometimes less and sometimes more	contra_past12mo_groupallSterilization	0.75	0.3	1.86	0.5352	0.6841
Sometimes less and sometimes more	disease_before_binary_num	1.36	1.05	1.76	0.0187	0.0495

Table S6

Pooled Multivariate Models (Periods Stopped)_LONGCOVID

term	PR	LowCI	HighCI	p.value	q.value
(Intercept)	0.04	0.04	0.05	0	0
age_scaled	0.79	0.74	0.86	0	0
bmi_groupObese	1.44	1.22	1.7	0	1e-04
bmi_groupOverweight	1.11	0.92	1.35	0.2664	0.305
bmi_groupUnderweight	1.38	0.88	2.15	0.1596	0.2305
covid_groupAcute COVID	1.15	0.97	1.37	0.1073	0.1743
covid_groupLong COVID	1.39	1.13	1.7	0.0015	0.0033
contra_past12mo_groupallCombined estrogen-progestin	1.26	1.03	1.55	0.026	0.0483
contra_past12mo_groupallCopper IUD	0.82	0.57	1.19	0.305	0.305
contra_past12mo_groupallOther	0.71	0.37	1.36	0.2998	0.305
contra_past12mo_groupallProgestin only	2.79	2.39	3.25	0	0
contra_past12mo_groupallSterilization	1.4	0.81	2.41	0.2262	0.2941
disease_before_binary_num	1.4	1.21	1.62	0	0

Table S7. Number of daily Long COVID symptoms across the menstrual cycle

	Number of distinct symptoms		
Predictors	*Incidence Rate Ratios*	*CI*	*p*
(Intercept)	12.19	10.05 – 14.80	**<0.001**
phase all [LS/M]	1.03	0.99 – 1.07	0.148
phase all [P]	1.00	0.95 – 1.05	0.878
age group [40 years+]	1.03	0.79 – 1.32	0.849
**Random Effects**			
σ^2	0.07		
$\tau_{00\ phase\_all:id}$	0.00		
$\tau_{00\ id}$	0.21		
ICC	0.75		
N_{phase_all}	3		
N_{id}	54		
Observations	1333		
Marginal R^2 / Conditional R^2	0.001 / 0.755		

Table S8: Adjusted models for the presence of long COVID symptoms across the menstrual cycle phases.

Symptom	Term	Estimate	StdError	ZValue	PValue	AdjustedPValue	OddsRatio	CI_Lower	CI_Upper
**Brain fog**	**(Intercept)**	**5.387**	**1.266**	**4.255**	**0**	**0**	**218.547**	**18.277**	**2613.276**
Brain fog	phase_allLS/M	0.1	0.232	0.431	0.666	0.822	1.105	0.701	1.741
Brain fog	phase_allP	-0.085	0.336	-0.251	0.802	0.899	0.919	0.475	1.775
Brain fog	age_groupBelow 40 years	-1.518	1.347	-1.127	0.26	0.539	0.219	0.016	3.071
**Memory issues**	**(Intercept)**	**2.926**	**0.903**	**3.24**	**0.001**	**0.008**	**18.653**	**3.178**	**109.495**
Memory issues	phase_allLS/M	-0.021	0.219	-0.094	0.925	0.958	0.979	0.637	1.504
Memory issues	phase_allP	-0.272	0.338	-0.806	0.42	0.69	0.762	0.393	1.478
Memory issues	age_groupBelow 40 years	-0.624	1.281	-0.487	0.626	0.807	0.536	0.044	6.598
**Speech issues**	**(Intercept)**	**2.001**	**0.736**	**2.72**	**0.007**	**0.043**	**7.396**	**1.748**	**31.298**
Speech issues	phase_allLS/M	0.286	0.185	1.544	0.123	0.332	1.331	0.926	1.913
Speech issues	phase_allP	-0.222	0.288	-0.769	0.442	0.699	0.801	0.455	1.408
Speech issues	age_groupBelow 40 years	-1.153	1.064	-1.084	0.278	0.556	0.316	0.039	2.541
Sensorimotor issues	(Intercept)	-0.435	0.547	-0.795	0.426	0.69	0.647	0.222	1.891
Sensorimotor issues	phase_allLS/M	0.287	0.159	1.806	0.071	0.206	1.332	0.976	1.82
Sensorimotor issues	phase_allP	-0.001	0.231	-0.002	0.998	0.998	0.999	0.635	1.571
Sensorimotor issues	age_groupBelow 40 years	0.227	0.811	0.28	0.779	0.899	1.255	0.256	6.151
Dizziness	(Intercept)	0.756	0.592	1.276	0.202	0.442	2.13	0.667	6.796
Dizziness	phase_allLS/M	0.41	0.162	2.522	0.012	0.063	1.507	1.097	2.07
Dizziness	phase_allP	0.083	0.245	0.337	0.736	0.88	1.087	0.672	1.756
Dizziness	age_groupBelow 40 years	-0.644	0.884	-0.728	0.467	0.7	0.525	0.093	2.97
Smell/Taste change	(Intercept)	-2.681	1.349	-1.987	0.047	0.16	0.068	0.005	0.964
Smell/Taste change	phase_allLS/M	-0.168	0.284	-0.59	0.555	0.766	0.845	0.484	1.475
Smell/Taste change	phase_allP	0.179	0.453	0.394	0.694	0.839	1.196	0.492	2.906
Smell/Taste change	age_groupBelow 40 years	-0.876	1.913	-0.458	0.647	0.816	0.416	0.01	17.699
Insomnia	(Intercept)	-0.375	0.516	-0.727	0.467	0.7	0.687	0.25	1.89
Insomnia	phase_allLS/M	-0.304	0.163	-1.862	0.063	0.198	0.738	0.536	1.016
Insomnia	phase_allP	-0.165	0.238	-0.694	0.488	0.715	0.848	0.532	1.352
Insomnia	age_groupBelow 40 years	0.182	0.766	0.238	0.812	0.899	1.2	0.267	5.384
Headache	(Intercept)	0.36	0.416	0.864	0.388	0.654	1.433	0.634	3.239
Headache	phase_allLS/M	0.384	0.154	2.49	0.013	0.066	1.468	1.086	1.985
Headache	phase_allP	0.237	0.225	1.053	0.292	0.561	1.267	0.815	1.97
Headache	age_groupBelow 40 years	0.27	0.626	0.432	0.666	0.822	1.31	0.384	4.468
**Disturbed sleep**	**(Intercept)**	**1.266**	**0.446**	**2.84**	**0.005**	**0.034**	**3.547**	**1.48**	**8.501**
Disturbed sleep	phase_allLS/M	-0.279	0.152	-1.832	0.067	0.205	0.757	0.562	1.019
Disturbed sleep	phase_allP	-0.303	0.216	-1.406	0.16	0.361	0.739	0.484	1.128
Disturbed sleep	age_groupBelow 40 years	-0.173	0.652	-0.266	0.79	0.899	0.841	0.234	3.019
**Fatigue**	**(Intercept)**	**7.937**	**1.725**	**4.601**	**0**	**0**	**2798.951**	**95.202**	**82289.599**
Fatigue	phase_allLS/M	-0.461	0.319	-1.444	0.149	0.353	0.631	0.337	1.178
Fatigue	phase_allP	-0.487	0.462	-1.054	0.292	0.561	0.614	0.248	1.52
Fatigue	age_groupBelow 40 years	0.443	1.453	0.305	0.76	0.899	1.557	0.09	26.866
**Post-exertional malaise**	**(Intercept)**	**5.906**	**1.809**	**3.265**	**0.001**	**0.008**	**367.234**	**10.595**	**12729.024**
Post-exertional malaise	phase_allLS/M	-0.488	0.268	-1.821	0.069	0.205	0.614	0.363	1.038
Post-exertional malaise	phase_allP	0.091	0.441	0.207	0.836	0.899	1.095	0.461	2.6
Post-exertional malaise	age_groupBelow 40 years	2.396	1.627	1.472	0.141	0.348	10.979	0.453	266.379
**Chills/Sweats**	**(Intercept)**	**-2.3**	**0.467**	**-4.922**	**0**	**0**	**0.1**	**0.04**	**0.25**
**Chills/Sweats**	**phase_allLS/M**	**0.831**	**0.173**	**4.81**	**0**	**0**	**2.296**	**1.635**	**3.222**
Chills/Sweats	phase_allP	0.598	0.25	2.393	0.017	0.082	1.818	1.114	2.968
Chills/Sweats	age_groupBelow 40 years	1.008	0.671	1.502	0.133	0.335	2.74	0.736	10.208
**Elevated temperature**	**(Intercept)**	**-4.283**	**0.84**	**-5.097**	**0**	**0**	**0.014**	**0.003**	**0.072**
Elevated temperature	phase_allLS/M	0.559	0.255	2.189	0.029	0.116	1.749	1.061	2.883
Elevated temperature	phase_allP	-0.667	0.441	-1.512	0.13	0.335	0.513	0.216	1.218
Elevated temperature	age_groupBelow 40 years	-0.217	1.041	-0.208	0.835	0.899	0.805	0.105	6.193
Heart palpitations	(Intercept)	-0.334	0.684	-0.489	0.625	0.807	0.716	0.187	2.736
Heart palpitations	phase_allLS/M	0.167	0.182	0.921	0.357	0.618	1.182	0.827	1.688
Heart palpitations	phase_allP	-0.082	0.28	-0.292	0.771	0.899	0.921	0.532	1.595
Heart palpitations	age_groupBelow 40 years	0.413	1.026	0.403	0.687	0.839	1.511	0.202	11.29
Tachycardia	(Intercept)	-0.607	0.842	-0.721	0.471	0.7	0.545	0.105	2.839
Tachycardia	phase_allLS/M	-0.029	0.204	-0.143	0.886	0.943	0.971	0.651	1.449
Tachycardia	phase_allP	-0.351	0.322	-1.087	0.277	0.556	0.704	0.375	1.323
Tachycardia	age_groupBelow 40 years	1.277	1.273	1.003	0.316	0.582	3.586	0.296	43.47
Chest pain	(Intercept)	-1.121	0.688	-1.629	0.103	0.291	0.326	0.085	1.255
Chest pain	phase_allLS/M	-0.154	0.179	-0.862	0.389	0.654	0.857	0.604	1.218
Chest pain	phase_allP	-0.217	0.288	-0.752	0.452	0.699	0.805	0.458	1.415
Chest pain	age_groupBelow 40 years	0.999	1.025	0.975	0.33	0.589	2.716	0.364	20.247
Chest tightness	(Intercept)	0.032	0.66	0.048	0.962	0.97	1.033	0.283	3.764
Chest tightness	phase_allLS/M	0.096	0.181	0.53	0.596	0.786	1.101	0.772	1.57
Chest tightness	phase_allP	-0.183	0.29	-0.632	0.527	0.746	0.833	0.472	1.47
Chest tightness	age_groupBelow 40 years	0.535	0.988	0.542	0.588	0.784	1.707	0.246	11.84
**Muscle aches**	**(Intercept)**	**1.986**	**0.681**	**2.917**	**0.004**	**0.029**	**7.286**	**1.918**	**27.681**
Muscle aches	phase_allLS/M	-0.012	0.186	-0.062	0.95	0.967	0.988	0.686	1.423
Muscle aches	phase_allP	-0.061	0.268	-0.229	0.819	0.899	0.941	0.556	1.591
Muscle aches	age_groupBelow 40 years	0.583	0.972	0.6	0.548	0.766	1.791	0.267	12.039
Joint pain	(Intercept)	1.676	0.829	2.022	0.043	0.16	5.344	1.052	27.135
Joint pain	phase_allLS/M	0.469	0.209	2.245	0.025	0.107	1.598	1.061	2.408
Joint pain	phase_allP	0.693	0.302	2.293	0.022	0.098	2	1.106	3.614
Joint pain	age_groupBelow 40 years	-1.095	1.17	-0.936	0.349	0.613	0.335	0.034	3.314
**Sore throat**	**(Intercept)**	**-1.76**	**0.47**	**-3.747**	**0**	**0**	**0.172**	**0.068**	**0.432**
Sore throat	phase_allLS/M	-0.035	0.168	-0.206	0.837	0.899	0.966	0.695	1.342
Sore throat	phase_allP	-0.528	0.264	-2.002	0.045	0.16	0.59	0.352	0.989
Sore throat	age_groupBelow 40 years	-0.047	0.7	-0.067	0.947	0.967	0.954	0.242	3.762
Vision issues	(Intercept)	-1.576	0.795	-1.983	0.047	0.16	0.207	0.044	0.982
**Vision issues**	**phase_allLS/M**	**0.734**	**0.214**	**3.423**	**0.001**	**0.008**	**2.083**	**1.37**	**3.169**
Vision issues	phase_allP	0.594	0.303	1.957	0.05	0.166	1.811	1	3.28
Vision issues	age_groupBelow 40 years	-1.483	1.176	-1.262	0.207	0.445	0.227	0.023	2.275
Tinnitus	(Intercept)	-3.43	1.316	-2.606	0.009	0.052	0.032	0.002	0.427
Tinnitus	phase_allLS/M	-0.389	0.243	-1.601	0.109	0.301	0.678	0.421	1.091
Tinnitus	phase_allP	-0.492	0.352	-1.398	0.162	0.361	0.611	0.307	1.219
Tinnitus	age_groupBelow 40 years	2.929	1.915	1.529	0.126	0.332	18.709	0.438	798.233
Breathlessness	(Intercept)	0.074	0.713	0.103	0.918	0.958	1.077	0.266	4.356
Breathlessness	phase_allLS/M	-0.086	0.185	-0.465	0.642	0.816	0.918	0.639	1.319
**Breathlessness**	**phase_allP**	**-0.759**	**0.283**	**-2.679**	**0.007**	**0.043**	**0.468**	**0.269**	**0.815**
Breathlessness	age_groupBelow 40 years	0.581	1.071	0.543	0.587	0.784	1.788	0.219	14.587
**Dry cough**	**(Intercept)**	**-1.861**	**0.518**	**-3.597**	**0**	**0**	**0.156**	**0.056**	**0.429**
Dry cough	phase_allLS/M	0.268	0.189	1.413	0.158	0.361	1.307	0.903	1.894
Dry cough	phase_allP	-0.815	0.374	-2.177	0.029	0.116	0.443	0.213	0.921
Dry cough	age_groupBelow 40 years	-1.66	0.812	-2.045	0.041	0.159	0.19	0.039	0.934
**Breathing issues**	**(Intercept)**	**-2.723**	**0.938**	**-2.902**	**0.004**	**0.029**	**0.066**	**0.01**	**0.413**
Breathing issues	phase_allLS/M	0.056	0.213	0.263	0.793	0.899	1.058	0.697	1.606
Breathing issues	phase_allP	-0.037	0.333	-0.111	0.912	0.958	0.964	0.502	1.851
Breathing issues	age_groupBelow 40 years	1.488	1.317	1.129	0.259	0.539	4.428	0.335	58.517
**Diarrhea**	**(Intercept)**	**-3.144**	**0.463**	**-6.796**	**0**	**0**	**0.043**	**0.017**	**0.107**
Diarrhea	phase_allLS/M	0.294	0.204	1.443	0.149	0.353	1.342	0.9	2.001
Diarrhea	phase_allP	0.231	0.291	0.793	0.428	0.69	1.26	0.712	2.229
Diarrhea	age_groupBelow 40 years	0.651	0.622	1.047	0.295	0.561	1.917	0.567	6.489
**Loss of appetite**	**(Intercept)**	**-2.817**	**0.592**	**-4.758**	**0**	**0**	**0.06**	**0.019**	**0.191**
Loss of appetite	phase_allLS/M	0.464	0.182	2.553	0.011	0.061	1.59	1.113	2.272
Loss of appetite	phase_allP	0.268	0.275	0.973	0.33	0.589	1.307	0.763	2.241
Loss of appetite	age_groupBelow 40 years	0.612	0.804	0.761	0.447	0.699	1.844	0.381	8.916
**Nausea**	**(Intercept)**	**-2.658**	**0.566**	**-4.698**	**0**	**0**	**0.07**	**0.023**	**0.213**
Nausea	phase_allLS/M	0.331	0.177	1.869	0.062	0.198	1.392	0.984	1.97
Nausea	phase_allP	0.179	0.276	0.65	0.515	0.738	1.196	0.696	2.054
Nausea	age_groupBelow 40 years	1.807	0.786	2.298	0.022	0.098	6.092	1.305	28.433
**Abdominal pain**	**(Intercept)**	**-3.524**	**0.813**	**-4.332**	**0**	**0**	**0.029**	**0.006**	**0.145**
Abdominal pain	phase_allLS/M	0.156	0.227	0.686	0.493	0.715	1.169	0.749	1.824
Abdominal pain	phase_allP	0.197	0.345	0.571	0.568	0.775	1.218	0.619	2.395
Abdominal pain	age_groupBelow 40 years	1.114	1.081	1.03	0.303	0.567	3.047	0.366	25.35

Table S9. Unadjusted models for severity of daily long COVID symptoms across the menstrual cycle phases.

Symptom	Term	Estimate	StdError	ZValue	PValue	AdjustedPValue	OddsRatio	CI_Lower	CI_Upper
Brain fog	Minor|Moderate	0.413	0.399	1.037	0.3	0.411	1.511	0.691	3.304
**Brain fog**	**Moderate|Severe**	**3.501**	**0.419**	**8.347**	**0**	**0**	**33.149**	**14.582**	**75.357**
Brain fog	phase_allLS/M	0.267	0.155	1.717	0.086	0.145	1.306	0.964	1.77
Brain fog	phase_allP	-0.07	0.226	-0.31	0.757	0.839	0.932	0.599	1.452
**Memory issues**	**Minor|Moderate**	**1.222**	**0.47**	**2.601**	**0.009**	**0.018**	**3.394**	**1.351**	**8.527**
**Memory issues**	**Moderate|Severe**	**4.63**	**0.522**	**8.864**	**0**	**0**	**102.514**	**36.851**	**285.18**
Memory issues	phase_allLS/M	0.105	0.188	0.561	0.575	0.696	1.111	0.768	1.606
Memory issues	phase_allP	-0.083	0.27	-0.306	0.759	0.839	0.92	0.542	1.562
**Speech issues**	**Minor|Moderate**	**1.572**	**0.393**	**3.995**	**0**	**0**	**4.816**	**2.229**	**10.405**
**Speech issues**	**Moderate|Severe**	**4.311**	**0.433**	**9.964**	**0**	**0**	**74.515**	**31.891**	**174.109**
Speech issues	phase_allLS/M	0.026	0.191	0.139	0.89	0.89	1.026	0.706	1.492
Speech issues	phase_allP	-0.068	0.271	-0.251	0.802	0.84	0.934	0.549	1.589
**Sensorimotor issues**	**Minor|Moderate**	**1.804**	**0.272**	**6.634**	**0**	**0**	**6.074**	**3.564**	**10.351**
**Sensorimotor issues**	**Moderate|Severe**	**4.995**	**0.421**	**11.864**	**0**	**0**	**147.673**	**64.705**	**337.026**
Sensorimotor issues	phase_allLS/M	0.325	0.231	1.407	0.16	0.252	1.384	0.88	2.177
Sensorimotor issues	phase_allP	-0.191	0.33	-0.578	0.563	0.689	0.826	0.433	1.577
**Dizziness**	**Minor|Moderate**	**1.847**	**0.345**	**5.353**	**0**	**0**	**6.341**	**3.225**	**12.468**
**Dizziness**	**Moderate|Severe**	**4.921**	**0.408**	**12.048**	**0**	**0**	**137.14**	**61.641**	**305.112**
**Dizziness**	**phase_allLS/M**	**0.664**	**0.214**	**3.099**	**0.002**	**0.005**	**1.943**	**1.277**	**2.955**
Dizziness	phase_allP	0.604	0.294	2.055	0.04	0.072	1.829	1.028	3.255
**Smell/Taste change**	**Minor|Moderate**	**8.673**	**1.924**	**4.507**	**0**	**0**	**5843.002**	**134.553**	**253733.555**
**Smell/Taste change**	**Moderate|Severe**	**12.207**	**2.095**	**5.826**	**0**	**0**	**200185.557**	**3297.105**	**12154378.096**
Smell/Taste change	phase_allLS/M	0.143	0.427	0.335	0.738	0.832	1.154	0.5	2.664
Smell/Taste change	phase_allP	0.296	0.585	0.507	0.612	0.726	1.344	0.427	4.232
Insomnia	Minor|Moderate	0.43	0.262	1.643	0.1	0.167	1.537	0.92	2.569
**Insomnia**	**Moderate|Severe**	**2.592**	**0.289**	**8.985**	**0**	**0**	**13.356**	**7.58**	**23.534**
Insomnia	phase_allLS/M	0.218	0.194	1.123	0.261	0.363	1.244	0.85	1.819
Insomnia	phase_allP	-0.069	0.294	-0.233	0.815	0.844	0.933	0.525	1.661
**Disturbed sleep**	**Minor|Moderate**	**0.569**	**0.24**	**2.376**	**0.017**	**0.033**	**1.766**	**1.104**	**2.828**
**Disturbed sleep**	**Moderate|Severe**	**2.556**	**0.259**	**9.883**	**0**	**0**	**12.884**	**7.755**	**21.405**
Disturbed sleep	phase_allLS/M	0.117	0.161	0.724	0.469	0.586	1.124	0.82	1.541
Disturbed sleep	phase_allP	0.181	0.238	0.761	0.447	0.571	1.198	0.752	1.911
**Fatigue**	**Minor|Moderate**	**-1.11**	**0.352**	**-3.15**	**0.002**	**0.005**	**0.33**	**0.165**	**0.657**
**Fatigue**	**Moderate|Severe**	**1.754**	**0.356**	**4.929**	**0**	**0**	**5.778**	**2.876**	**11.609**
**Fatigue**	**phase_allLS/M**	**0.585**	**0.134**	**4.379**	**0**	**0**	**1.795**	**1.38**	**2.334**
Fatigue	phase_allP	0.175	0.188	0.931	0.352	0.46	1.191	0.824	1.722
**Post-exertional malaise**	**Minor|Moderate**	**-1.101**	**0.357**	**-3.083**	**0.002**	**0.005**	**0.333**	**0.165**	**0.669**
**Post-exertional malaise**	**Moderate|Severe**	**1.742**	**0.361**	**4.829**	**0**	**0**	**5.709**	**2.814**	**11.583**
**Post-exertional malaise**	**phase_allLS/M**	**0.37**	**0.14**	**2.647**	**0.008**	**0.016**	**1.448**	**1.1**	**1.905**
**Post-exertional malaise**	**phase_allP**	**0.487**	**0.194**	**2.509**	**0.012**	**0.024**	**1.627**	**1.113**	**2.38**
**Chills/Sweats**	**Minor|Moderate**	**1.461**	**0.336**	**4.346**	**0**	**0**	**4.31**	**2.231**	**8.327**
**Chills/Sweats**	**Moderate|Severe**	**3.816**	**0.422**	**9.047**	**0**	**0**	**45.422**	**19.863**	**103.868**
Chills/Sweats	phase_allLS/M	0.35	0.282	1.24	0.215	0.307	1.419	0.817	2.466
Chills/Sweats	phase_allP	0.23	0.477	0.482	0.63	0.732	1.259	0.494	3.206
**Elevated temperature**	**Minor|Moderate**	**1.973**	**0.339**	**5.822**	**0**	**0**	**7.192**	**3.701**	**13.977**
**Elevated temperature**	**Moderate|Severe**	**4.206**	**0.468**	**8.99**	**0**	**0**	**67.088**	**26.809**	**167.885**
Elevated temperature	phase_allLS/M	0.127	0.508	0.249	0.803	0.84	1.135	0.42	3.073
NA	NA	NA	NA	NA	NA	NA	NA	NA	NA
**Heart palpitations**	**Minor|Moderate**	**1.115**	**0.413**	**2.701**	**0.007**	**0.015**	**3.05**	**1.357**	**6.852**
**Heart palpitations**	**Moderate|Severe**	**3.823**	**0.448**	**8.528**	**0**	**0**	**45.741**	**19.009**	**110.066**
Heart palpitations	phase_allLS/M	-0.072	0.209	-0.344	0.731	0.832	0.931	0.618	1.402
Heart palpitations	phase_allP	-0.32	0.285	-1.121	0.262	0.363	0.726	0.415	1.269
Tachycardia	Minor|Moderate	1.049	0.526	1.994	0.046	0.081	2.855	1.018	8.004
**Tachycardia**	**Moderate|Severe**	**3.997**	**0.562**	**7.117**	**0**	**0**	**54.435**	**18.092**	**163.779**
Tachycardia	phase_allLS/M	-0.038	0.203	-0.186	0.853	0.861	0.963	0.647	1.433
Tachycardia	phase_allP	-0.196	0.267	-0.732	0.464	0.586	0.822	0.487	1.387
**Chest pain**	**Minor|Moderate**	**0.695**	**0.303**	**2.295**	**0.022**	**0.041**	**2.004**	**1.106**	**3.629**
**Chest pain**	**Moderate|Severe**	**4.011**	**0.374**	**10.738**	**0**	**0**	**55.202**	**26.522**	**114.897**
Chest pain	phase_allLS/M	-0.262	0.212	-1.238	0.216	0.307	0.77	0.508	1.166
Chest pain	phase_allP	0.286	0.284	1.004	0.315	0.423	1.331	0.763	2.322
**Chest tightness**	**Minor|Moderate**	**1.363**	**0.438**	**3.115**	**0.002**	**0.005**	**3.908**	**1.656**	**9.221**
**Chest tightness**	**Moderate|Severe**	**5.175**	**0.498**	**10.388**	**0**	**0**	**176.797**	**66.614**	**469.224**
Chest tightness	phase_allLS/M	0.297	0.209	1.421	0.155	0.248	1.346	0.893	2.027
Chest tightness	phase_allP	0.501	0.279	1.796	0.073	0.125	1.65	0.955	2.851
Muscle aches	Minor|Moderate	0.497	0.395	1.26	0.208	0.303	1.644	0.758	3.565
**Muscle aches**	**Moderate|Severe**	**3.547**	**0.422**	**8.405**	**0**	**0**	**34.709**	**15.179**	**79.37**
**Muscle aches**	**phase_allLS/M**	**0.39**	**0.168**	**2.318**	**0.02**	**0.038**	**1.477**	**1.063**	**2.053**
Muscle aches	phase_allP	0.347	0.251	1.383	0.167	0.256	1.415	0.865	2.314
Joint pain	Minor|Moderate	0.58	0.425	1.364	0.172	0.26	1.786	0.776	4.108
**Joint pain**	**Moderate|Severe**	**3.127**	**0.447**	**6.993**	**0**	**0**	**22.805**	**9.496**	**54.769**
Joint pain	phase_allLS/M	0.163	0.18	0.905	0.366	0.473	1.177	0.827	1.675
Joint pain	phase_allP	0.277	0.279	0.99	0.322	0.426	1.319	0.764	2.279
**Vision issues**	**Minor|Moderate**	**2.497**	**0.599**	**4.169**	**0**	**0**	**12.146**	**3.755**	**39.293**
**Vision issues**	**Moderate|Severe**	**7.077**	**0.841**	**8.42**	**0**	**0**	**1184.41**	**227.839**	**6157.093**
Vision issues	phase_allLS/M	0.093	0.346	0.27	0.787	0.84	1.097	0.557	2.162
Vision issues	phase_allP	-0.203	0.501	-0.406	0.685	0.788	0.816	0.306	2.179
**Breathlessness**	**Minor|Moderate**	**1.331**	**0.492**	**2.703**	**0.007**	**0.015**	**3.785**	**1.443**	**9.928**
**Breathlessness**	**Moderate|Severe**	**6.505**	**0.622**	**10.466**	**0**	**0**	**668.476**	**197.528**	**2262.261**
Breathlessness	phase_allLS/M	0.128	0.234	0.549	0.583	0.698	1.137	0.718	1.798
Breathlessness	phase_allP	0.509	0.344	1.481	0.139	0.225	1.664	0.848	3.265
**Dry cough**	**Minor|Moderate**	**2.48**	**0.654**	**3.794**	**0**	**0**	**11.941**	**3.314**	**43.028**
**Dry cough**	**Moderate|Severe**	**4.105**	**0.738**	**5.561**	**0**	**0**	**60.643**	**14.275**	**257.619**
Dry cough	phase_allLS/M	0.737	0.456	1.617	0.106	0.174	2.09	0.855	5.108
Dry cough	phase_allP	0.261	0.999	0.261	0.794	0.84	1.298	0.183	9.198
**Breathing issues**	**Minor|Moderate**	**2.164**	**0.767**	**2.821**	**0.005**	**0.011**	**8.706**	**1.936**	**39.147**
**Breathing issues**	**Moderate|Severe**	**7.413**	**0.949**	**7.808**	**0**	**0**	**1657.391**	**258**	**10647.07**
Breathing issues	phase_allLS/M	0.431	0.311	1.386	0.166	0.256	1.539	0.836	2.831
**Breathing issues**	**phase_allP**	**1.145**	**0.427**	**2.678**	**0.007**	**0.015**	**3.142**	**1.361**	**7.257**
**Headache**	**Minor|Moderate**	**0.959**	**0.22**	**4.361**	**0**	**0**	**2.609**	**1.695**	**4.016**
**Headache**	**Moderate|Severe**	**3.375**	**0.26**	**13.003**	**0**	**0**	**29.224**	**17.556**	**48.647**
**Headache**	**phase_allLS/M**	**0.746**	**0.175**	**4.257**	**0**	**0**	**2.109**	**1.496**	**2.971**
**Headache**	**phase_allP**	**1.032**	**0.237**	**4.346**	**0**	**0**	**2.807**	**1.764**	**4.466**
**Loss of appetite**	**Minor|Moderate**	**1.512**	**0.315**	**4.801**	**0**	**0**	**4.536**	**2.446**	**8.41**
**Loss of appetite**	**Moderate|Severe**	**3.71**	**0.417**	**8.899**	**0**	**0**	**40.854**	**18.042**	**92.51**
Loss of appetite	phase_allLS/M	-0.315	0.314	-1.003	0.316	0.423	0.73	0.394	1.35
Loss of appetite	phase_allP	0.214	0.432	0.495	0.621	0.729	1.239	0.531	2.888
**Nausea**	**Minor|Moderate**	**1.85**	**0.297**	**6.224**	**0**	**0**	**6.36**	**3.553**	**11.383**
**Nausea**	**Moderate|Severe**	**3.783**	**0.381**	**9.924**	**0**	**0**	**43.948**	**20.827**	**92.736**
Nausea	phase_allLS/M	0.386	0.292	1.322	0.186	0.278	1.471	0.83	2.607
Nausea	phase_allP	0.811	0.375	2.164	0.03	0.055	2.25	1.079	4.693
**Sore throat**	**Minor|Moderate**	**1.648**	**0.496**	**3.32**	**0.001**	**0.002**	**5.197**	**1.966**	**13.738**
**Sore throat**	**Moderate|Severe**	**4.083**	**0.578**	**7.061**	**0**	**0**	**59.323**	**19.108**	**184.174**
Sore throat	phase_allLS/M	0.197	0.304	0.649	0.516	0.638	1.218	0.671	2.21
Sore throat	phase_allP	-0.915	0.5	-1.829	0.067	0.117	0.401	0.15	1.067
**Diarrhea**	**Minor|Moderate**	**1.923**	**0.537**	**3.579**	**0**	**0**	**6.841**	**2.388**	**19.6**
**Diarrhea**	**Moderate|Severe**	**3.455**	**0.614**	**5.625**	**0**	**0**	**31.658**	**9.503**	**105.471**
Diarrhea	phase_allLS/M	-0.116	0.422	-0.276	0.783	0.84	0.89	0.389	2.036
Diarrhea	phase_allP	0.122	0.643	0.19	0.849	0.861	1.13	0.32	3.984
**Abdominal pain**	**Minor|Moderate**	**1.142**	**0.3**	**3.808**	**0**	**0**	**3.133**	**1.74**	**5.641**
**Abdominal pain**	**Moderate|Severe**	**3.188**	**0.397**	**8.027**	**0**	**0**	**24.24**	**11.133**	**52.779**
Abdominal pain	phase_allLS/M	0.055	0.296	0.184	0.854	0.861	1.057	0.591	1.887
Abdominal pain	phase_allP	-0.723	0.552	-1.311	0.19	0.28	0.485	0.164	1.432
**Tinnitus**	**Minor|Moderate**	**3.266**	**0.993**	**3.29**	**0.001**	**0.002**	**26.206**	**3.742**	**183.512**
**Tinnitus**	**Moderate|Severe**	**8.085**	**1.152**	**7.019**	**0**	**0**	**3245.42**	**339.366**	**31036.525**
**Tinnitus**	**phase_allLS/M**	**0.852**	**0.359**	**2.37**	**0.018**	**0.034**	**2.344**	**1.16**	**4.738**
Tinnitus	phase_allP	0.157	0.553	0.284	0.776	0.84	1.17	0.396	3.459

Table S10. TaqMan qPCR Primers and UPL probes

Gene	Forward Primer Sequence	Reverse Primer Sequence	Probe
ATP5B	agaggtcccatcaaaaccaa	tcctgctcaacactcatttcc	50
SHDA	tccactacatgacggagcag	ccatcttcagttctgctaaacg	20
ESR1	aaccagtgcaccattgataaaa	tcctcttcggtcttttcgtatc	69
AR	gccttgctctctagcctcaa	ggtcgtccacgtgtaagttg	14
PGR	tttaagagggcaatggaagg	cggattttatcaacgatgcag	11
PRB	ggagacgagatctcctaacaattact	cttggcctccatcctgtc	5
IFNG	ggcattttgaagaattggaaag	tttggatgctctggtcatctt	21
IL-6	gatgagtacaaaagtcctgatcc	ctgcagccactggttctgt	40
IL-8	agacagcagagcacacaagc	atggttccttccggtggt	72
IL-10	tgggggagaacctgaagac	ccttgctcttgttttcacagg	30
TNF	tccagacttccttgagacacg	cccggtctcccaaataaatac	36

Table S11. Immunohistochemistry Methods and Reagents

Target Protein	Antibody	Type	Conc.	Refine Protocol	Epitope Retrieval
Androgen Receptor/AR	Abcam ab133273	Rabbit monoclonal	470ρg/µl	Mse 60’, 15, 15	HIER 2 PH9 20’
Cathelicidin/ CLP	Abcam ab69484	Rabbit polyclonal	1.3ng/µl	60’, 15, no pp	HIER 1 PH6 20’
CD68	Dako M0814	Mouse monoclonal	410ρg/µl	Mse 60’, 15, 15	HIER 1 PH6 20’
Progesterone Receptor/PG	Dako M3569	Mouse monoclonal	160ρg/µl	Mse 60’, 15, 15	HIER 1 PH6 20’
