# Supplementary material for: The potential bidirectional relationship between long COVID and menstruation
Source: Nat Commun. 2025 Sep 16;16:8187. doi: 10.1038/s41467-025-62965-7 (PMC12441152; doi:10.1038/s41467-025-62965-7)
Supplement: Supplementary file 2 — Reporting Summary [file 41467_2025_62965_MOESM2_ESM.pdf]

nature portfolio

Corresponding author(s): Jacqueline Maybin

Last updated by author(s): Jul 26, 2025

Reporting Summary

Nature Portfolio wishes to improve the reproducibility of the work that we publish. This form provides structure for consistency and transparency in reporting. For further information on Nature Portfolio policies, see our Editorial Policies and the Editorial Policy Checklist.

Statistics

For all statistical analyses, confirm that the following items are present in the figure legend, table legend, main text, or Methods section.

n/a	Confirmed	
☐	☒	The exact sample size (*n*) for each experimental group/condition, given as a discrete number and unit of measurement
☐	☒	A statement on whether measurements were taken from distinct samples or whether the same sample was measured repeatedly
☐	☒	The statistical test(s) used AND whether they are one- or two-sided<br>*Only common tests should be described solely by name; describe more complex techniques in the Methods section.*
☐	☒	A description of all covariates tested
☐	☒	A description of any assumptions or corrections, such as tests of normality and adjustment for multiple comparisons
☐	☒	A full description of the statistical parameters including central tendency (e.g. means) or other basic estimates (e.g. regression coefficient) AND variation (e.g. standard deviation) or associated estimates of uncertainty (e.g. confidence intervals)
☐	☒	For null hypothesis testing, the test statistic (e.g. *F*, *t*, *r*) with confidence intervals, effect sizes, degrees of freedom and *P* value noted<br>*Give P values as exact values whenever suitable.*
☒	☐	For Bayesian analysis, information on the choice of priors and Markov chain Monte Carlo settings
☒	☐	For hierarchical and complex designs, identification of the appropriate level for tests and full reporting of outcomes
☒	☐	Estimates of effect sizes (e.g. Cohen's *d*, Pearson's *r*), indicating how they were calculated

Our web collection on statistics for biologists contains articles on many of the points above.

Software and code

Policy information about availability of computer code

Data collection	Qualtrics platform (www.qualtrics.com)<br>Balance App (https://www.balance-menopause.com/balance-app/)
Data analysis	R Studio<br>Graphpad Prism 10.4.1

For manuscripts utilizing custom algorithms or software that are central to the research but not yet described in published literature, software must be made available to editors and reviewers. We strongly encourage code deposition in a community repository (e.g. GitHub). See the Nature Portfolio guidelines for submitting code & software for further information.

Data

Policy information about availability of data

All manuscripts must include a data availability statement. This statement should provide the following information, where applicable:

- Accession codes, unique identifiers, or web links for publicly available datasets
- A description of any restrictions on data availability
- For clinical datasets or third party data, please ensure that the statement adheres to our policy

De-identified survey and app data generated in this study (Figures 1 and 3) have been deposited on GitHub https://github.com/ataquette/Long-COVID-Mens.

Source data for Figures 4 and 5 are provided with this paper. Any additional information required to reanalyse the data reported in this paper is available from the authors upon reasonable request.

Research involving human participants, their data, or biological material

Policy information about studies with human participants or human data. See also policy information about sex, gender (identity/presentation), and sexual orientation and race, ethnicity and racism.

Reporting on sex and gender	This manuscript reports changes in menstrual parameters in people who menstruated. We have not included male subjects as they do not menstruate.
Reporting on race, ethnicity, or other socially relevant groupings	Details provided in methods section.
Population characteristics	Provided in methods and results section and Table 1 and 2.
Recruitment	Details for each study provided in methods section
Ethics oversight	Detailed for each study in methods section.<br>"The COVID-19 Pandemic and Women's Reproductive Health" received a favourable ethical opinion from the Oxford University School of Anthropology and Museum Ethnography Departmental Research Ethics Committee [SME_C1A_20_029].<br>The prosepective long COVID symptom study was reviewed by CNRS and received GDPR regulation approval (DPD 2022/10).<br>The biological study received a favourable ethical opinion from East Midlands-Leicester South Research Ethics Committee (21/EM/0166).

Note that full information on the approval of the study protocol must also be provided in the manuscript.

Field-specific reporting

Please select the one below that is the best fit for your research. If you are not sure, read the appropriate sections before making your selection.

☒ Life sciences ☐ Behavioural & social sciences ☐ Ecological, evolutionary & environmental sciences

For a reference copy of the document with all sections, see nature.com/documents/nr-reporting-summary-flat.pdf

Life sciences study design

All studies must disclose on these points even when the disclosure is negative.

Sample size	Our power calculation estimated that comparing n=10 samples from women with Long COVID and n=20 sample from controls at a single time point would give 80% power to detect as significant a true mean difference of approximately 1.2 standard deviations.
Data exclusions	All excluded participants are noted in Figure 2 and any exclusions in analysis are fully disclosed in the methods section, with justification.
Replication	Findings cannot be reproduced due to the nature of the survey data. Biological sample assessment cannot be reproduced due to limited human samples. Details of sample processing in duplicate/triplicate are included in relevant methods section.
Randomization	It was not possible to randomise samples/participants. Details of consideration of covariates detailed in manuscript.
Blinding	It was not possible to blind researchers at time of group allocation.

Reporting for specific materials, systems and methods

We require information from authors about some types of materials, experimental systems and methods used in many studies. Here, indicate whether each material, system or method listed is relevant to your study. If you are not sure if a list item applies to your research, read the appropriate section before selecting a response.

Materials & experimental systems

n/a	Involved in the study	
☐	☒	Antibodies
☒	☐	Eukaryotic cell lines
☒	☐	Palaeontology and archaeology
☒	☐	Animals and other organisms
☐	☒	Clinical data
☒	☐	Dual use research of concern
☒	☐	Plants

Methods

n/a	Involved in the study	
☒	☐	ChIP-seq
☒	☐	Flow cytometry
☒	☐	MRI-based neuroimaging

Antibodies

Antibodies used

Detailed in table S11

Validation

AR ab133272 tested on human IHC-P For unpurified use at 1/100-1/250.
CLP/LL-37 ab69484 was tested on human IHC-P.
CD68 m0814 he antibody was clustered as anti-CD68 at the Fourth International Workshop and Conference on Human Leucocyte Differentiation Antigens held in Vienna in 1989. DS-PAGE analysis of immunoprecipitates formed between the antibody and 125I-labeled lysates from human spleen with B-cell lymphoma rich in macrophages shows reaction with a 110 kDa polypeptide, corresponding to CD68.In Western blotting of extracts of lung, spleen and U937 cells, diffuse 110, 70 and 40 kDa bands were detected when using reducing conditions. Under non-reducing conditions the spleen extract showed an additional 220 kDa band (4).
PR Dako M3569 Anti-PR, PgR 636 has been demonstrated to react with the PR-A and PR-B forms by Western biot of whole Geil extracts and reaGts with both free and hormone-bound PR.' The epitope has been mapped tG the amino terminai domain shared by PR-A and PR-B.

Clinical data

Policy information about clinical studies
All manuscripts should comply with the ICMJE guidelines for publication of clinical research and a completed CONSORT checklist must be included with all submissions.

Clinical trial registration

21/EM/0166

Study protocol

https://www.hra.nhs.uk/planning-and-improving-research/application-summaries/research-summaries/ergo-study/

Data collection

Included in methods.

Outcomes

Details in methods. Outcome measures = markers of ovarian function and markers of inflammation.

Plants

Seed stocks

N/A

Novel plant genotypes

N/A

Authentication

N/A
